# Biotransformation of cinnamyl alcohol to rosavins by non-transformed wild type and hairy root cultures of *Rhodiola kirilowii*

**DOI:** 10.1007/s10529-013-1401-5

**Published:** 2013-11-05

**Authors:** Marta Grech-Baran, Katarzyna Sykłowska-Baranek, Anna Krajewska-Patan, Anna Wyrwał, Agnieszka Pietrosiuk

**Affiliations:** 1 Department of Biology and Pharmaceutical Botany, Faculty of Pharmacy, Medical University of Warsaw, Banacha 1 St., 02-097 Warsaw, Poland; 2 Institute of Natural Fibres and Medicinal Plants, Libelta 27 St., 61-707 Poznan, Poland

**Keywords:** Biotransformation, Cinnamyl alcohol, Hairy root culture, Non-transformed wild type root culture, *Rhodiolakirilowii*, Rosavin, Rosarin

## Abstract

Non-transformed wild type (NTWT) and hairy root cultures of *Rhodiola kirilowii* were grown in medium supplemented with 2.5 mM cinnamyl alcohol as a precursor and/or sucrose (1 %) on the day of inoculation or on the 14th day of culture. Rosarin, rosavin, and rosin were produced by NTWT root culture but only rosarin and rosavin by hairy roots. Approximately 80 and 95 % of the glycosides were released into the medium for NTWT and hairy root cultures, respectively. The highest rosavin yield, 505 ± 106 mg/l, was in hairy root culture when cinnamyl alcohol was applied on the day of inoculation with the addition of sucrose on the 14th day of culture. For rosin production, supplementation with cinnamyl alcohol alone on day 14 was more favourable with the highest amount 74 ± 10 mg/l in NTWT root culture. Only traces of rosarin were detected.

## Introduction

**Fig. 1:**
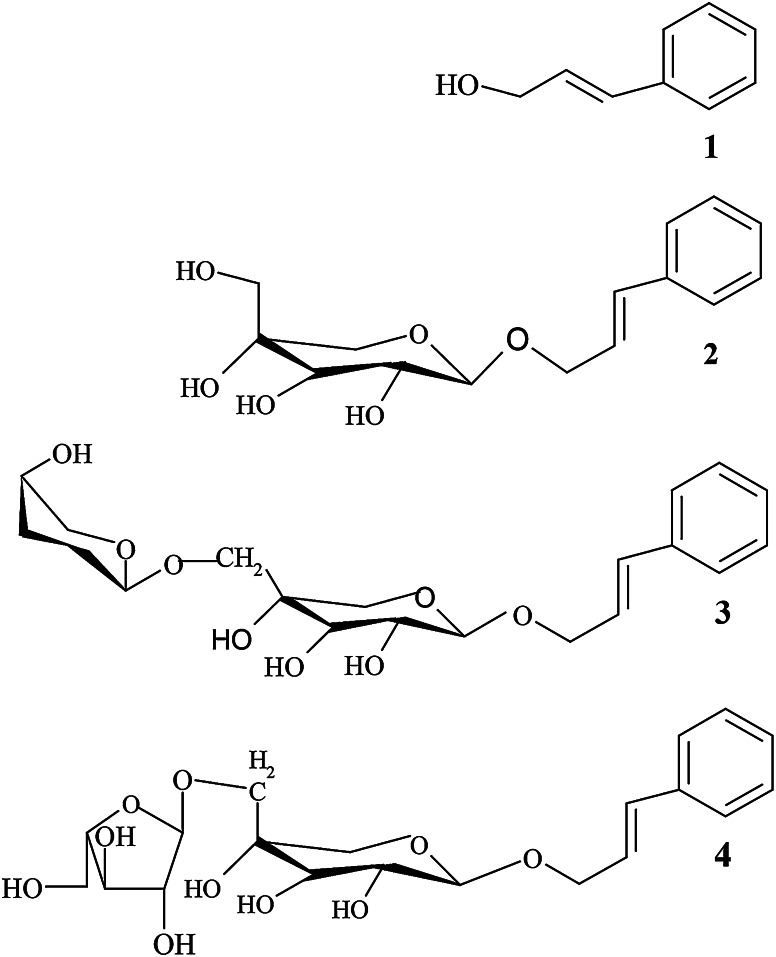
Chemical structures of cinnamyl alcohol glycosides. Cinnamyl alcohol (*1*), rosin (*2*), rosavin (*3*), and rosarin (*4*)

*Rhodiola kirilowii* (Crassulaceae) is a medicinal plant that grows wild in Asia and Eastern Europe. Its roots and rhizomes contain over 49 different bioactive chemicals (Krajewska-Patan et al. 2008). Among these, the most important are salidroside—a tyrosol glycoside and cinnamyl alcohol glycosides, including rosavin, rosin and rosarin (Fig. 1), usually referred to as rosavins. Different biological activities are attributed to these compounds, such as adaptogenic (Panossian and Wikman 2009), antiviral (Zuo et al. 2007), neuroprotective (Yu et al. 2010), immunity-increasing (Wójcik et al. 2009), and antitumor activities (Zdanowski et al. 2012).

**Fig. 2:**
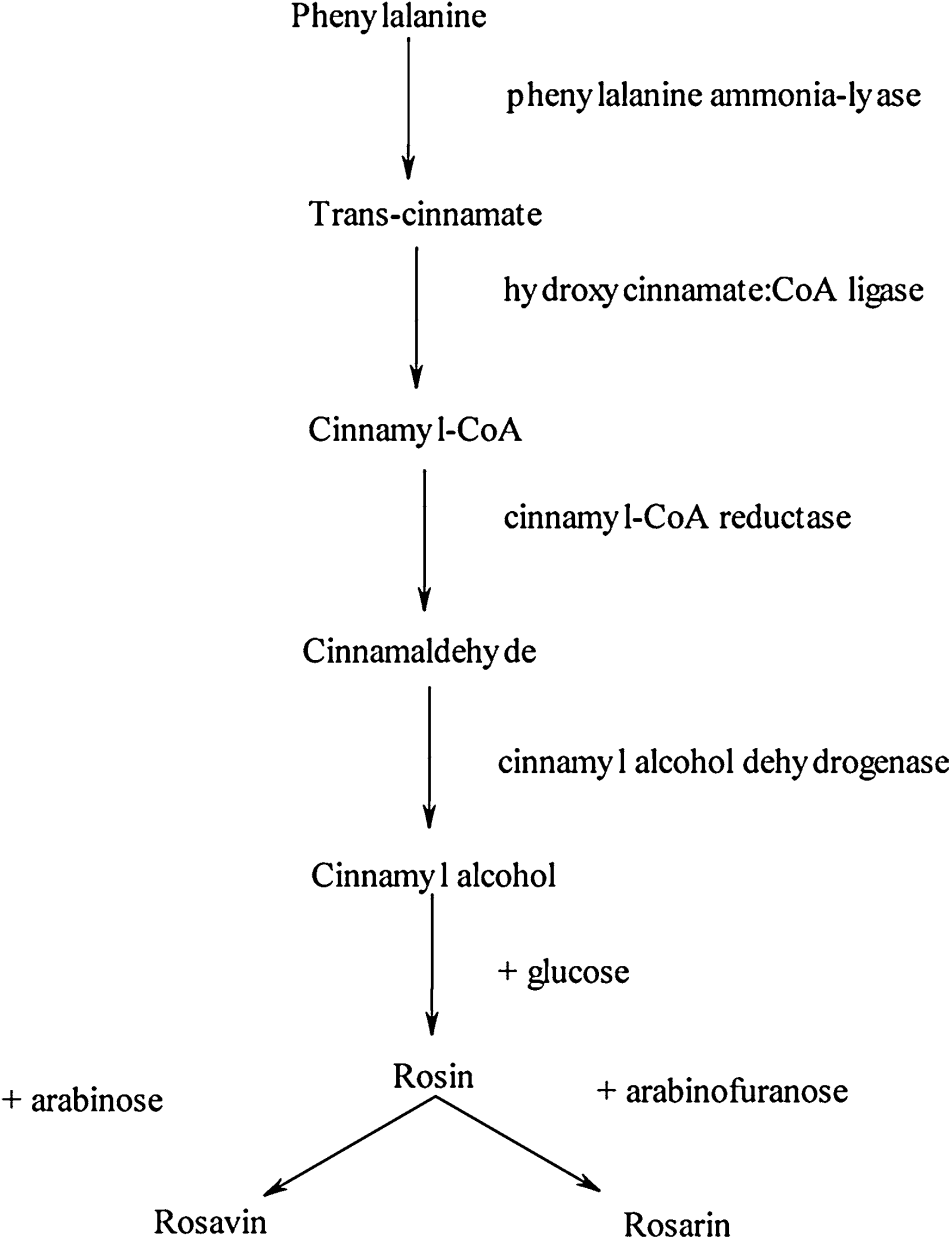
Biosynthetic pathway of rosin, rosavin and rosarin (György 2006)

Rosavin production in *Rhodiola* is restricted to only a few genera. Biosynthesis (Fig. 2) of phenolic glycosides occurs spontaneously in *Rhodiola* roots and rhizomes (Krajewska-Patan et al. 2013). Therefore, our study focused on the glycosylation of trans-cinnamyl alcohol into rosavins by the non-transformed wild type (NTWT) and hairy root cultures. To date, hairy root cultures are preferred over plant cell/callus and suspension cultures owing to their genetic/biochemical stability, hormone autotrophy, multienzyme biosynthetic potential (representing that of the parent plants), and relatively low-cost cultural requirements (Banerjee et al. 2012).

Biotransformation process is useful for enhancing and extending the production of desirable products and is independent of the kind of plant culture used (Giri et al. 2003; Shilpa et al. 2010). Moreover, biotransformation allows changes to phytomolecules that are not realistically attainable by chemical semisynthesis (Banerjee et al. 2012). The content of salidroside in *Rhodiola* spp. has been enhanced (Krajewska-Patan et al. 2007a; Xu et al. 1998a, b; Wu et al. 2003; Zhou et al. 2007) but only a few papers (Furmanowa et al. 1999; György et al. 2004, 2005; Krajewska-Patan et al. 2007b) have discussed the biotransformation of cinnamyl alcohol to rosavins.

To the best of our knowledge, this is the first study aimed at developing a satisfactory strategy for significantly enhancing rosavin production in NTWT and hairy *R.* *kirilowii* root cultures. Moreover, this report is the first to show that cinnamyl glycosides are mostly released into the culture medium.

## Materials and methods

### Experimental materials

#### Non-transformedwild type (NTWT) root culture

Seeds of *R. kirilowii* were collected from the Medicinal Plant Garden of the Institute of Natural Fibres and Medicinal Plants in Poznan, Poland. They were germinated in vitro on Linsmaier and Skoog solid medium (Linsmaier and Skoog 1965) without growth regulators, consisting of 3 % (w/v) sucrose and 0.78 % (w/v) agar, in the dark at 24 °C. Then shoot tips were excised from the in vitro-germinated seedlings and rooted on LS medium supplemented with 10 mg adenine sulfate/l, 1 ml kinetin/l, 5 ml indole-3-butyric acid/l, 3 % (w/v) sucrose, and solidified with 0.78 % (w/v) agar. NTWT root culture was established from roots derived from 1-month-old plantlets. The roots were cultured in 20 ml LS liquid medium supplemented with 10 mg adenine sulphate/l, 1 mg kinetin/l, and 5 mg indole-3-butyric acid/l with shaking at 105 rpm and 24 °C in the dark. Every 4 weeks, 0.4 ± 0.07 g of fresh weight of roots was transferred into a fresh LS medium.

#### Hairy root culture

Hairy roots were established by infection of the *R. kirilowii* plantlets with *Agrobacterium rhizogenes* LBA 9402 (Zych et al. 2008) that had been grown in 50 ml YEB solid medium (Vervilet et al. 1975) for 72 h at 24 °C in the dark at 105 rpm. The cultures was diluted (1:4) with YEB liquid medium before transformation. The plantlets were directly wounded with sterile needles containing the bacterial suspension. Hairy roots appeared 11 days after initial infection. Each root that emerged from the place of infection was excised and cultivated as a separate hairy root line. The transformation process was confirmed by PCR. DNA analysis showed that *rol B* gene and aux 1 gene from Ri plasmid *of A. rhizogenes* were incorporated into the plant genome of *R. kirilowii.*

The best growing transformed root clone (RKTR1) was used. Hairy roots culture were grown in Erlenmeyer flasks containing 20 ml modified DCR medium (Gupta and Durzan 1985) without phytohormones at 105 rpm in the dark at 24 °C. Every 4 weeks, 0.4 ± 0.06 g of roots was subcultured on to fresh DCR medium.

### Growth kinetics experiments

Biomass growth curves for the NTWT and hairy root cultures were determined in parallel. From day 3 through day 27, 3 flasks were collected from each treatment every 3 days. The roots were washed with deionized water and blotted dry. Next, the roots were lyophilized, and the dry weight (DW) was recorded. The root biomass increase was expressed as the ratio of final weight to initial weight. Each experiment was performed in triplicate.

### Enhancement of rosavin production

To enhance rosavin production, a cinnamyl alcohol (CA) and/or sucrose feeding strategy was applied. Four types of experiments were performed: (1) CA was added to the medium on the day of inoculation to give 2.5 mM; (2) CA (2.5 mM) was added to the medium on the day of inoculation and the medium was further supplemented with 1 %, (w/v) sucrose on the 14th day of culture; (3) CA (2.5 mM) was added to the medium on the 14th day of culture; (4) sucrose (1 %) and CA (2.5 mM) were both added on the 14th day of culture.

NTWT and hairy root cultures growing in unsupplemented medium were used as control cultures. Each experiment was performed in triplicate. Ten flasks were taken for each type of culture at the end of the 28 day growth cycle.

### Chemical analysis

The extraction method of Wiedenfeld et al. (2007) was applied to lyophilized roots and post-culture media used for extraction. Root samples were sonicated in methanol using a Baldin Sonorex system. Extraction was performed three times for 30 min at 40 °C. The extracts were evaporated, and the dry residue was partitioned between carbon tetrachloride/methanol/water (5:4:1, by vol.). The methanol/water extract was evaporated to dryness, and the residue was dissolved in n-butanol/water, separated into two fractions, and evaporated. Lyophilized post-culture media were dissolved in methanol, filtered, and evaporated. The steps that followed were the same as those used for root analysis. The dry residue from each fraction was dissolved in methanol and analysed by HPLC using a C18 column and gradient eluting with acetonitrile/phosphoric acid/methanol (from 7:85:8 to 20:60:20, by vol.) at 1 ml/min over 30 min. The eluent was monitored at 205, 220, 250, and 275 nm. Cinnamyl alcohol, rosarin, rosavin, and rosin were used as standards and were analysed under the same conditions. Peaks were assigned by spiking the samples with standards and by comparison of the retention times and UV spectra. Mean (*n* = 30) and ± standard error were calculated for each experiment. The results were analysed using STATISTICA PL 10.

### Statistical analysis

The data obtained from each experiment were analysed using the Kruskal–Wallis and Tukey tests. The differences between analysed natural and hairy root cultures were compared using the Mann–Whitney test. *P* < *0.05* was considered to indicate statistically significant differences.

## Results and discussion

### Non-transformed wild type (NTWT) and hairy root growth

NTWT and hairy roots varied in their growth requirements with respect to the mineral composition of the applied LS or DCR media. For cultivation of NTWT roots, LS medium proved the most suitable while no biomass increase was observed in DCR medium. In contrast, DCR medium promoted the best growth of hairy roots (data not shown).

**Fig. 3:**
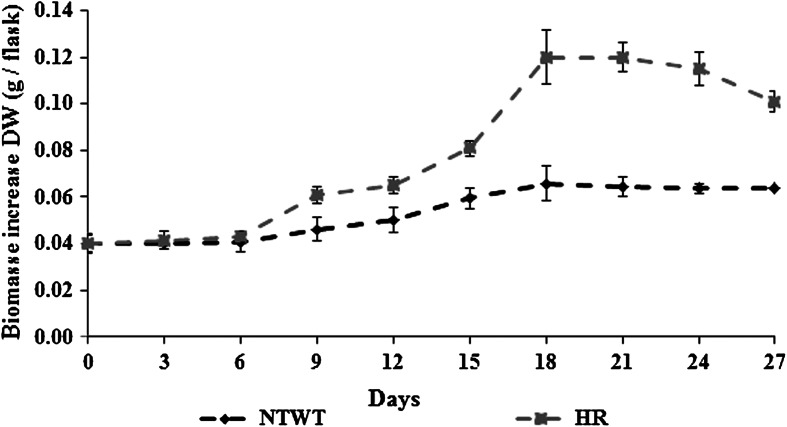
Dry biomass increase in *R. kirilowii* non-transformed wild type (NTWTR) and hairy (HR) root cultures. Non-transformed wild type roots were cultivated in LS liquid medium (Linsmaier and Skoog 1965) supplemented with 10 mg/l adenine sulphate, 1 mg/l kinetin, and 5 mg/l indole-3-butyric acid. Hairy roots were cultured in modified hormone free DCR liquid medium (Gupta and Durzan 1985)

In both hairy and NTWT root control cultures the stationary growth phase began on the 18th day of culture (Fig. 3). Hairy roots had higher fresh and dry growth rates (final/inoculum) than NTWT roots (58 and 54 %, respectively. As measurement of the dry weight was more accurate than that of the fresh weight, this was used in subsequent work.

Cinnamyl alcohol (CA) adversely affected growth hairy and NTWT root. Hairy root culture fed with CA on the day of inoculation showed a 55 % growth inhibition in comparison to control while those fed the CA on the 14th day after inoculation showed a 44 % decrease. The addition of CA on the day of inoculation and sucrose on day 14 of culture resulted in 57 % growth decrease. Supplementation with CA and sucrose on the day 14 caused a 38 % fall in biomass accumulation.

Growth of NTWT root culture was inhibited by 48 and 40 % in comparison to the control when CA was added on the day of inoculation or on the 14th day of the growth cycle, respectively. When the medium was fed with CA on the day of inoculation and sucrose on day 14 culture root growth was reduced by 51 and by 37 % when CA and sucrose were added to the medium together on day 14.

The correlation between biomass increase and the biotransformation reaction has been discussed by György et al. (2004) in compact callus aggregate culture. The decrease in biomass growth was from 20 to 50 % when CA was at 0.05 or 5 mM, respectively. In a further study, György et al. (2005) observed that addition of both glucose and sucrose to the culture medium did not improve the accumulation of compact callus aggregate tissue biomass. Our results showed that supplying additional sucrose to the culture medium did not have a significant effect on the growth of NTWT and hairy root cultures of *R. kirilowii.*

### Production of cinnamyl alcohol glycosides

CA and rosavins were not detected in NTWT and hairy root cultures maintained in control media. In all the experiments, the addition of exogenous CA to the media induced the production of cinnamyl alcohol glycosides. However, significant variation was observed in the amounts of the investigated compounds detected, in accordance with the type of cultivated roots and the experimental strategy used.

Phytochemical analyses of the NTWT roots showed the presence of all three rosavins, with the highest being rosin at 3 ± 0.4 mg/g DW (Table 1). The rosavin yield was 2.6 ± 0.3 mg/g DW, while the rosarin yield 0.09 ± 0.02 mg/g DW. The addition of sucrose alone to the culture on day 14 after inoculation increased production of rosavins (Table 1). Rosin production was 40 % higher (5 ± 0.7 mg/g DW) relative to the level noted in the first experiment. Increases in rosavin and rosarin concentration reached 3.4 ± 0.8 and 0.12 ± 0.04 mg/g DW, respectively. The highest conversion of exogenous CA to rosavin by NTWT root culture (6.4 ± 0.4 mg/g) was when the roots were fed with CA on day 14 (Table 1). However, this led to lower amounts of rosarin and rosin 0.07 ± 0.01 and 4.6 ± 1 mg/g, respectively. Medium supplementation with both CA and sucrose on day 14 inhibited rosin and rosarin production. The accumulation of rosin (3.4 ± 1 mg/g DW) was 34 % lower in comparison to its level when CA was added on the day of inoculation followed by sucrose addition on the 14th day but was still higher than when CA was added to the medium on the inoculation day with no further supplementation (Table 1). The rosavin yield (6 ± 0.5 mg/g DW) was not affected by simultaneous CA and sucrose addition on day 14. Statistical analyses showed significant differences in rosavin production in roots between each of the experimental conditions, with the highest production (*p* = 0.0147) obtained when CA was added on day 14. The rosarin or rosin content was not significantly affected by the applied feeding strategy in NTWT root cultures.

**Table 1:** Intracellular (mg/g DW) content of cinnamyl alcohol glycosides in *R. kirilowii* non-transformed wild type and hairy root cultures

Experiment type	Rosarin	Rosavin	Rosin
Root line			
NTWT			
Control	n.d.	n.d.	n.d.
1	0.09 ± 0.02	2.6 ± 0.3	3 ± 0.4
2	0.12 ± 0.04	3.4 ± 0.8	5 ± 0.7
3	0.07 ± 0.01	6.4 ± 0.4	4.6 ± 1
4	0.01 ± 0.01	6 ± 0.5	3.4 ± 1
HR			
Control	n.d.	n.d.	n.d.
1	0.4 ± 0.07	3.4 ± 0.6	n.d.
2	0.3 ± 0.1	6.7 ± 0.7	n.d.
3	0.06 ± 0.01	9.8 ± 0.2	n.d.
4	0.08 ± 0.02	5.4 ± 2	n.d.

Data represents means of three repetitions ± SD. Types of experiments: control (unsupplemented media); 1, after addition of exogenous CA on the inoculation day; 2, after addition of exogenous CA on the inoculation day and addition of sucrose on day 14; 3, after addition of exogenous CA on day 14; 4, after addition of exogenous CA and sucrose on day 14. Rosavins content were determined at the end of 28-day growth cycle

*n.d.* not detected, *NTWTR* non-transformed wild type roots, *HR* hairy roots

Most of the rosavin and rosin (80 %) was released to the medium in NTWT root cultures (Table 2). However, rosarin was not detected in the postculture media for any of the experimental conditions. The highest rosavin concentration (42 ± 15 mg/l) was detected in postculture media when CA was added on the inoculation day and additional sucrose was provided on day 14 (Table 2), whereas rosin addition of CA on day 14 (66 ± 20 mg/ml; Table 2). The highest intra- and extracellular rosavin concentrations were achieved when CA was added at the beginning of culture, and sucrose added on day 14. The culture requirements for rosin production differed from those for rosavin, with the maximum total rosin accumulation occurring with the addition of CA on day 14.

**Table 2:** The total content (mg/l) of cinnamyl alcohol glycosides in *R. kirilowii* non-transformed wild type and hairy root cultures

Experiment type	Rosarin			Rosavin				Rosin		
	Intra-cellular	Extra-cellular	Total	Intra-cellular	Extra-cellular	Total	Intra-cellular		Extra-cellular	Total
Root line										
NTWT										
Control	n.d	n.d.	n.d	n.d	n.d	n.d	n.d		n.d	n.d
1	0.2 ± 0.01	n.d	0.2 ± 0.01	5 ± 0.8	37 ± 12	42 ± 12	5 ± 1		28 ± 6	33 ± 9
2	0.2 ± 0.1	n.d	0.2 ± 0.1	7 ± 1	42 ± 15	49 ± 11	12 ± 3		37 ± 9	49 ± 10
3	0.15 ± 0.04	n.d	0.15 ± 0.04	14 ± 1	11 ± 3	25 ± 3	8 ± 2		66 ± 20	74 ± 10
4	0.1 ± 0.04	n.d	0.1 ± 0.04	14 ± 3	9 ± 2	23 ± 5	6 ± 1		22 ± 7	28 ± 10
HR										
Control	n.d.	n.d	n.d	n.d	n.d	n.d	n.d.		n.d	n.d
1	0.9 ± 0.1	n.d	0.9 ± 0.1	8 ± 1	272 ± 12	280 ± 10	n.d		n.d	n.d
2	0.7 ± 0.2	n.d.	0.7 ± 0.2	14 ± 3	491 ± 95	505 ± 106	n.d		n.d	n.d
3	0.1 ± 0.01	n.d.	0.1 ± 0.01	20 ± 2	353 ± 84	373 ± 95	n.d		n.d	n.d
4	0.14 ± 0.04	n.d	0.14 ± 0.04	12 ± 4	343 ± 71	355 ± 83	n.d		n.d	n.d

Data represents means of three repetitions ± SD. Types of experiments: control (unsupplemented media); 1, after addition of exogenous CA on the inoculation day; 2, after addition of exogenous CA on the inoculation day and addition of sucrose on day 14; 3, after addition of exogenous CA on day 14; 4, after addition of exogenous CA and sucrose on day 14. Rosavins content were determined at the end of 28-day growth cycle

*n.d.* not detected, *NTWTR* non-transformed wild type roots, *HR* hairy roots

In contrast to the results for NTWT root culture, no rosin production was observed in hairy root culture. CA supplementation on the inoculation day provided 3.4 ± 0.6 and 0.4 ± 0.07 mg/g DW rosavin and rosarin, respectively (Table 1). Addition of sucrose to the culture on day 14 led to a 50 % increase in rosavin production to 6.7 ± 0.7 mg/g DW, while the rosarin concentration was not affected (0.3 ± 0.1 mg/g DW). Under the third set of experimental conditions (addition of CA on day 14), the observed rosavin level was markedly high, reaching 9.8 ± 0.2 mg/g DW (Table 1); this was the highest yield of this glycoside detected in roots in all the experiments. Culture supplementation with both CA and sucrose on day 14 diminished rosavin accumulation in hairy roots by 45 % to 5.4 ± 2 mg/g DW. In contrast, the combined addition of CA and sucrose on day 14 increased the yield of rosarin to 0.08 ± 0.02 mg/g DW (*p* = 0.01).

In hairy root culture over 95 % of the rosavin cinnamyl glycoside content was released to the media (Table 2), whereas rosarin and rosin could not be detected in any of the postculture media. When exogenous CA was added on the inoculation day, extracellular rosavin reached 272 ± 12 mg/l that is 97 % higher than its intracellular yield (calculated as extracellular\intracellular content; Table 2). The extracellular rosavin content in hairy root cultures was also 87 % higher than the results of analyses of post culture media from NTWT root culture. When sucrose was added on day 14 the amount of rosavin in the media increased up to 491 ± 94 mg/l, that is 97 % higher than that in the roots and 91 % more than that in the postculture media of NTWT roots maintained under the same conditions (Table 2).

Similar to the results for NTWT root culture, the highest intra- and extra-cellular rosavin contents were detected when CA was added on the inoculation day, followed by sucrose addition on day 14 (Table 2). In contrast to the results for NTWT root culture, rosin production was not observed in the hairy root culture, while rosavin and rosarin production was higher. Statistical analyses showed highly significant differences between the precursor application strategy and/or sucrose feeding between hairy and NTWT types of culture.

The plant cells are factories producing a wide spectrum of secondary metabolites and the majority of them are considered as potent drug candidates. One of the biotechnological strategies, the biotransformation process has been developed. Until now, biotransformation has contributed to an increase of excepted rosavins in callus culture, suspension cell culture of *Rhodiola* spp. has been reported (Furmanowa et al. 1999; György et al. 2004, 2005; Krajewska-Patan et al. 2007b). Furmanowa et al. (1999) measured the glycosylation level of exogenously applied CA (2.5 mM) in *R. rosea* cell suspension culture. Their results indicated that 90 % of the CA used as the substrate for biotransformation was converted by the cells to a number of constituents, including rosavin, up to 1 % within 72 h. Our results showed that conversion of CA was over 94 % and 96 % for the NTWT and hairy root cultures, respectively.

In studies involving *R. rosea* compact callus aggregate liquid culture, György et al. (2004) observed that optimal concentration of CA was 2 mM for rosin production. It increased by 1.25 %. The highest rosavin, 0.083 % was in the presence of 0.1 mM CA. Moreover, no rosarin was detected. The data presented in our study show that for rosavin production in both NTWT and hairy root cultures, the most favourable concentration of CA was 2.5 mM.

In further studies, György et al. (2005) investigated the sucrose and/or glucose feeding. Rosavin was not produced at all when only sucrose was added but the rosin yield increased to 0.54 % when 10 g glucose and 2 mM CA were applied. Moreover, four new unexpected rosavin derivatives were detected. Nevertheless, the rosavin yield was still very low, reaching a maximum of 0.015 %.

Krajewska-Patan et al. (2007b) examined the biotransformation capacity of *R. rosea* compact callus aggregate culture. Compact callus aggregate maintained on solid medium after 2.5 mM CA addition produced the whole spectrum of active compounds present in the intact plant: salidroside, *p*-tyrosol, rosavins, and chlorogenic and gallic acids. However, the rosin and rosavin levels (1.06 and 0.15 % respectively) were significantly lower.

## Conclusions

*Rhodiola kirilowii* root cultures are capable of biotransformation reactions. Hairy *R. kirilowii* root cultures could be considered as a more potent and abundant source of rosavin than NTWT root cultures.

Without exogenous CA supplementation, none of the examined phenolic glycosides were formed in roots or secreted to the post culture media. Addition of CA enabled rosarin, rosavin, and rosin production in NTWT root cultures and rosarin and rosin production in hairy cultures. Moreover, we have demonstrated that feeding cultures with CA on the inoculation day and additional sucrose on day 14 of the growth cycle resulted in increased rosavin production. For rosin accumulation, the most advantageous strategy is the addition of CA alone on day 14. Rosarin was detected only in similar trace amounts in NTWT and hairy root cultures. Regardless of the experiment type, 80–95 % of glycosides were released to the media in NTWT and hairy root cultures. Taken together, the biotransformation process could constitute an effective tool in the stimulation of the important biologically active compounds in plant material obtained via biotechnological methods.
